# Glabridin Liposome Ameliorating UVB-Induced Erythema and Lethery Skin by Suppressing Inflammatory Cytokine Production

**DOI:** 10.4014/jmb.2011.11006

**Published:** 2021-02-02

**Authors:** Chijian Zhang, Yongjie Lu, Yong Ai, Xian Xu, Siyang Zhu, Bing Zhang, Minghui Tang, Lanyue Zhang, Tinggang He

**Affiliations:** 1 Hua An Tang Biotech Group Co., Ltd., Guangzhou 510000, P.R. China; 2 Guangdong He Ji Biotech Co., Ltd., Guangzhou 510000, P.R. China; 3 School of Biomedical and Pharmaceutical Sciences, Guangdong University of Technology, Guangzhou 510006, P.R. China

**Keywords:** Glabridin, glabridin liposome, skin photoaging, anti-inflammation

## Abstract

Glabridin, a compound of the flavonoid, has shown outstanding skin-whitening and anti-aging properties, but its water insolubility limits its wide application. Therefore, glabridin liposome (GL) has been developed to improve its poor bioavailability, while there are few studies to evaluate its amelioration of UVB- induced photoaging. This study is performed to investigate the amelioration of GL against UVB- induced cutaneous photoaging. The prepared GL has a spheroidal morphology with an average diameter of 200 nm. The GL shows lower cytotoxicity than glabridin, but it has a more effective role in inhibition of melanin. Moreover, the application of GL can effectively relieve UV radiation induced erythema and leathery skin, associated with the down-regulated expression of inflammatory cytokines (TNF-α, IL-6 and IL-10). Taken together, these results demonstrate that GL has potentials as topical therapeutic agents against UVB radiation induced skin damage through inhibiting inflammation.

## 1. Introduction

The human skin is the outer tissue and the largest organ in human body, which serves as a general defense system against the outside environmental aggressions [1]. Ultraviolet-B (UVB) radiation is one of the most harmful environmental factors induced biological damage on the skin. Repeated exposure to UV radiation disastrously causes premature skin aging or photoaging that is characterized by the formation of coarse wrinkles, erythema, edema, hyperplasia, inelasticity, dryness, laxity, pigmentation and other skin disorders [2-6].

UVB radiation induced reactive oxygen species (ROS) in human skin may activate mitogen-activated protein kinases (MAPKs), such as extracellular signal-regulated kinase (ERK), p38 kinase and c-Jun N-terminal kinase (JNK). The skin has developed a defense system against ROS, but the continuous formation of ROS disrupts its enzymatic and non-enzymatic anti-oxidant defense systems [7-9]. Furthermore, UVB radiation induced inflammatory response is a common phenomenon [10]. ROS generated from UV-B radiation may affect MAPK signaling and activate nuclear factor-κB and activation protein 1 to release inflammatory cytokines, including tumor necrosis factor-α (TNF-α), interleukin-1β (IL-1β) and interleukin-6 (IL-6). In addition, the increased matrix metalloproteinase (MMP) activity in response to ROS leads to the destruction of extracellular matrix (ECM) structure and function through collagen degradation [11]. Exposure to UVB radiation also activates the expression of cyclooxygenase-2 (COX-2), which aggravates skin inflammation by promoting the production of prostaglandin E2 and inducing the formation of inducible nitric oxide synthase (iNOS) [12-15]. Obviously, the down-regulated expression of inflammatory factors can effectively alleviate the photoaging of skin.

**Fig. 1:**
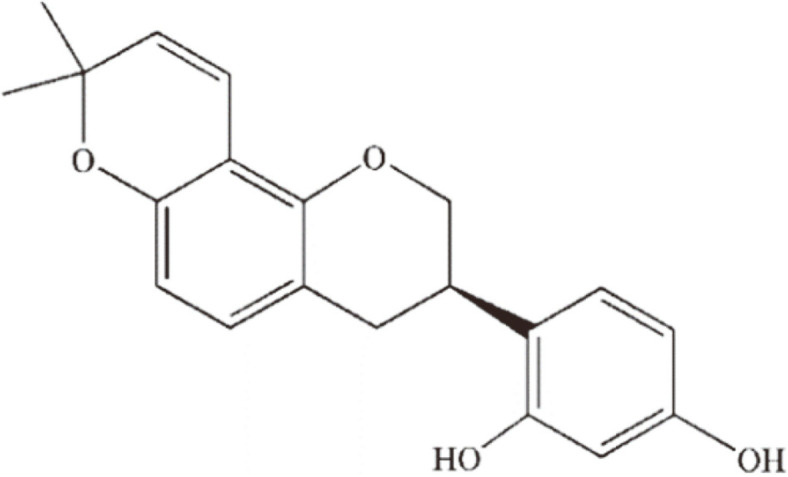
Chemical structure of glabridin.

Leguminosae is the third largest family of dicotyledon plants with about 690 genera and more than 17600 species. These plants are widely distributed throughout the world, with approximately 172 genera and 1485 species in China. The roots and rhizomes of *Glycyrrhiza glabra* L (family: Leguminosae) have been clinically used for centuries in antidotes, demulcents, expectorants and remedies for inflammation [16]. It is also usually used as natural flavoring, food and cosmetic additives [17]. Glabridin (Fig. 1), a species-specific isoflavan from roots of G. glabra, has been verified with multiple pharmacological actions, such as cardiovascular protective, anti-oxidant, anti-obesity, anti-microbial and anti-inflammatory activities [18-20]. Glabridin exhibits anti-inflammatory activity by inhibiting the level of pro-inflammatory cytokines and reducing the concentration of nitric oxide (NO) or ROS [13, 20], realized by the inhibition of dendritic cell maturation through blocking inflammatory related factors like NF-κB and MAPK signals, which plays an important role in regulating the immune system, especially in autoimmune diseases [21].

However, the water-insolubility of glabridin and the hydrophilicity of cuticle cell cause a barrier effect against the assimilation of glabridin in external preparations. Liposome is a microvesicle with a lipid-like bilayer structure, and can fuse with lipid bilayers in the cuticle cell, promote transdermal absorption of vesicle drugs and improve their bioavailability [22]. Thus far, most of the studies on glabridin are focused on purification and applications [23, 24]. There is a lack of systematic experiment to comparatively study the amelioration of glabridin (GB) and glabridin liposome (GL) on UVB radiation induced skin damage. In this study, GL is prepared by thin-film dispersed method and an animal model of skin photoaging induced by UVB radiation is established to evaluate their amelioration performance. It is expected to provide a theoretical basis for the application of GB and GL in sunscreen and anti-aging products.

## Materials and Methods

### Material and Chemicals

Glabridin (GB) purchased from Qinghai Lake Pharmaceutical Co., Ltd. (China). Soy lecithin, cholesterol, sodium stearate, butanediol, octanediol, and hexanediol purchased from Aladdin Biochem Co., Ltd. (China). Immunohistochemistry analysis kits (TNF-α, IL-10, and IL-6) provided by Absin Bioscience Inc (China). The hematoxylin-eosin staining kit for histological analysis provided by Beijing Solarbio Science & Technology Co., Ltd. (China). All chemicals were of analytical reagents and used without purification.

### Preparation of Glabridin Liposome

Glabridin liposome (GL) was prepared by film dispersion method followed the reported procedures with modification [25]. Soy lecithin (2 g), sodium stearate (0.2 g) and glabridin (0.3 g) were dissolved into hot methanol (50 ml), and then mixed with dichloromethane (20 ml) containing cholesterol (0.86 g). The resulting mixture was transferred into a round-bottom flask, and dried under vacuum at 65°C until a thin film formed. A solution consisting of water (68 g), butanediol (30 g) and octanediol/ hexanediol (2 g) was added, and maintained at 65°C until the film was dissolved into liquid suspension. Blank liposome (BL) was prepared by the same procedure without the addition of glabridin.

### Particle Size and Morphology

The particle size and zeta potential of GL were measured by a Zetasizer (NANO ZS, Malvern Instruments, UK). The morphology of GL was observed with a transmission electron microscope (JEM-2100, Jeol, Japan).

### Encapsulation Efficiency

**Fig. 2:**
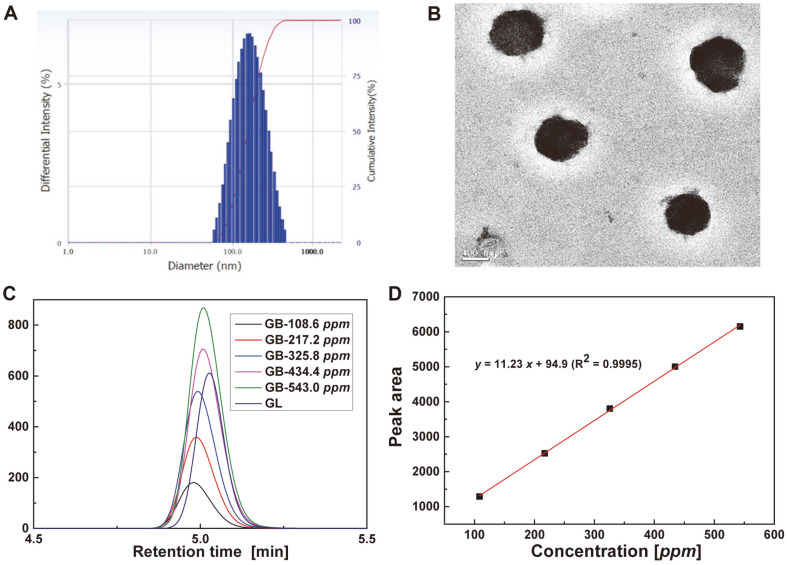
The physical properties of glabridin liposome. (**A**) size distribution, (**B**) TEM image, (**C**) HPLC curves, and (**D**) linear regression.

The content of glabridin was measured by Agilent 1260 HPLC with Zorbax SB-C_18_ chromatographic column (150 mm × 4.6 mm, 5 μm), acetonitrile with 2 % acetic acid (*v/v*) as mobile phase, DAD detector with a wavelength of 280 nm, flow rate of 1.0 ml/min, injection volume of 10 μl. A linear-regression analysis of glabridin content was performed from the known content versus its HPLC absorption peak areas (Fig. 2C). Therefore, the encapsulation efficiency (%) of glabridin was calculated after demulsification with methanol-water solution (9:1, *v/v*), by comparing the weight of glabridin inside the liposomes with that of the total feeding weight.

$$\mathrm{Encapsulation}\mathrm{efficiency}(\%)=W_{E}/W_{F}\times 100$$

where W_E_ and W_F_ were weight of encapsulated and total feed glabridin, respectively.

### Cytotoxicity

To evaluate the potential cytotoxicity of glabridin and its liposome, MTT (3-(4,5-dimethylthiazol-2-yl)-2,5-diphenyl-2H-tetrazolium bromide) colorimetric assay was carried out with B16 murine melanoma cell line (B16) and human keratinocyte cell line (HaCaT). The cells were seeded at a density of 5 × 10^3^ cell per well of a 96-well plate and treated with different concentrations of GB, GL and BL in 37°C incubator for 24 h with 5% CO_2_. Then, 20 μl of MTT solution (5 mg/ml) were added to each well and cells were kept at 37°C for 3 h. The supernatant was carefully aspirated, and its absorbance at 570 nm was measured by a microplate reader (Bio-Rad, USA). The inhibition percentage (*I*%) was determined using the following formula,

$$I\%=(\frac{A_{\mathrm{blank}}-A_{\mathrm{sample}}}{A_{\mathrm{blank}}})\times 100$$

where *A_blank_* was the absorbance of control solution without GB, GL and its blank liposome (BL), and *A_sample_* was the absorbance of sample solution.

### Melanin Content

The influence of GB, GL and BL on melanin relative content in B16 cells was estimated referring to the reported method with slight modification [26]. B16 cells were seeded in a 12-well plate with a density of 2 × 10^5^ cells per well and then incubated at 37°C with 5% CO_2_ for 24 h. After various concentration samples and 200 nM α-MSH added for 48 h, cells were lysed in 1 ml of 1 M NaOH (containing 10% DMSO) by ultrasound for 30 min, incubated at 80°C in water bath for 1 h, and centrifuged for 20 min by 3,000 rpm. Optical density (OD) of supernatant was measured at 475 nm by an ELISA reader. The protein content was determined by BCA method. Relative melanin content was calculated by the following equation:

$$\mathrm{Melanin}\mathrm{relative}\mathrm{content}(\%)={\mathrm{OD}}_{s}/{\mathrm{OD}}_{0}\times 100$$

where OD_0_ and OD_s_ were OD of control and sample, respectively.

### Animals Experiment

The female nude mice (6-wk old, about 15 g body weight) bought from the Guangdong Provincial Laboratory Animal Center (approval number: SCXK [Guangzhou]-2018-0020). The mice were acclimated at 22°C with a 12-h light-dark cycle for one week. GB, GL, and BL were dissolved in ethanol at a concentration of 20, 40, or 60 mg/ml for the later use. The mice were randomly divided into nine groups with three mice, including model group (MC), solvent group (SC), control group (CON) and six sample groups (GB or GL). GB/GL-L, GB/GL-M and GB/GL-H represented as the concentration of 20, 40, and 60 mg/ml, respectively. About 150 μl of various solutions was topically applied to the dorsal area every day for eight weeks.

### UVB Radiation

UVB radiation was implemented by a UV apparatus (China) with lamps at 285-350 nm wavelength. The radiation dose result in erythema on mice dorsal skin was defined as one minimal erythema dose (1 MED), which was calculated to be approximately 100 mJ/cm^2^. The radiation dose was increased by 1 Med per week, from 1 Med to 3 Med, and remained at 3 Med until the end of experiment. The mice were exposed to UVB radiation three times per week for eight weeks.

### Histological and Immunohistochemical Analysis

For histological analysis, the mice were sacrificed by cervical dislocation at the end of experiment, and the skin tissues were fixed with 4% formalin and embedded in paraffin. After dewaxing, the slice with 5 μm thickness was obtained. The sections were stained by hematoxylin and eosin (H&E) staining. The images were recorded by Olympus IX71 Digital Camera (Olympus, Japan) at 200× magnifications. The skin thickness was measured by digital analysis (ImageJ, USA), and the thickness referred to the distance from the basement membrane of the cell space to the bottom of cuticle.

The immunohistochemical analysis was performed as the previously reported method with modifications [27]. For antigen retrieval, 5-μm tissue sections were heated for 20 min at 100°C in 10 mM sodium citrate solution containing 0.05% Tween-20 (pH 6.0). The sections were then incubated with TNF-α, IL-10, or IL-6 antibodies (dilution 1:200) at 4°C overnight. After washing thrice with PBS, the sections were incubated with the secondary antibody and alkaline phosphatase-labeled streptavidin (1:200) h at 25°C for 1. Washing with PBS, the sections were stained with chromogenic reagent (3,30-diaminobenzidine) for 10 min, and washed thoroughly by distilled water. The treated tissue sections were mounted on slides and photographed under a fluorescence microscope (Olympus IX71) at 200× magnifications. The number of TNF-α, IL-10, or IL-6 positive cells in sample images was determined by virtue of Image-Pro Plus 6.0 software (Media Cybernetics, USA).

### Statistical Analysis

All quantitative data were presented as mean value ± standard deviation. The value variance among groups were analyzed by One-way ANOVA. The *p* < 0.05 considered to be statistically signiﬁcant. All analyses were performed using SPSS 17.0 statistical analysis software (IBM, USA).

## Results and Discussion

### Morphology and Size of Glabridin Liposome

To achieve effective encapsulation of GB, the popular film dispersion method is performed. Fig. 2A indicates that the average particle size of GL is about 200 nm and its polydispersity index (PDI) is 0.2, which means the liposome has uniform size distribution. The morphology of GL is also observed by TEM, and Fig. 2B revea ls that the liposome has spheroidal morphology with a size of 150~200 nm. The glabridin in liposome can be identified by HPLC (Fig. 2C), and thus its concentration (3003 ± 23 *ppm*) can be calculated from the linear regression in Fig. 2D. The encapsulation efficiency is thus calculated to be higher than 95%.

### Cytotoxicity

**Fig. 3:**
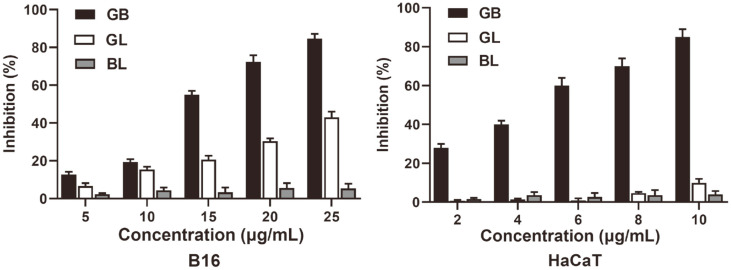
Cytotoxicity of glabridin, glabridin liposome and blank liposome.

To clearly understand the cytotoxicity, various dosages of GB, GL and BL are applied to B16 murine melanoma (B16) and human keratinocyte (HaCaT) cells. As shown in Fig. 3, the inhibition to B16 and HaCaT cells is gradually increased by the increasing concentration of GB, GL and BL, while the GB shows the highest inhibition and the BL has the lowest value at the same concentration. Particularly, the inhibition of GB exceeds 80% at 25 μg/ml, but it is lower than 50% and 10% for GL and BL to B16 cells. Regarding to HaCaT cells, the GB leads to 85%inhibition at a concentration of 10μg/ml, while the inhibition caused by GL and BL is less than 10%. Obviously, the application of liposome can effectively alleviate cytotoxicity of glabridin, because the liposomes can gradually release the encapsulated drugs and decrease its concentrations compared to free glabridin, which is aligned with the reported literature [28, 29].

### Melanin Content

**Fig. 4:**
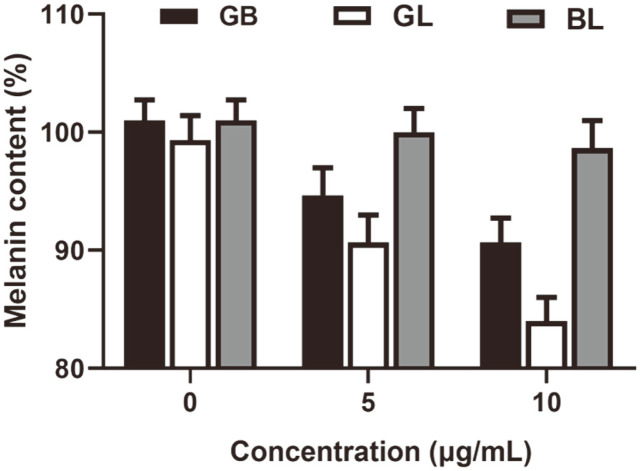
Melanin content of B16 cells after treated by various concentration of glabridin, glabridin liposome and blank liposome.

To evaluate the influence of GB, GL and BL on the production of melanin, various concentrations of GB, GL, and BL are also applied to B16 cells. Fig. 4 reveals that the relative content of melanin for BL treatment is very closed to the untreated controls. Contrarily, the melanin content gradually decreases with the increasing concentration of GB and GL, because they can inhibit the formation of melanin [30]. Moreover, the content for GL treatment is obviously lower than that of GB treatment at the same concentration, which result from the enhanced compatibility of liposome [31].

### Glabridin Liposome Reduced Skin Lesions Induced by UV-B Radiation

**Fig. 5:**
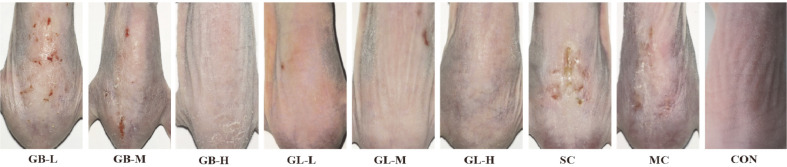
Representative photographs of UV-B-irradiated mice dorsal skin with different treatments.

In order to estimate their anti-photoaging activity, 150 μl GL and GB solution with different concentrations are topically applied to the back skin of hairless mice exposed to UV-B radiation. The representative skin images in Fig. 5 have clearly revealed that the mice of solvent group (SC) and model group (MC) compared with the untreated control (non-UV radiation) suffer from seriously rough and scaly skin, meaning that the solvent has no skin protective effect. In contrast, the topical application of GL or GB solutions can relieve UV radiation induced skin lesions such as erythema and leathery skin. Especially, the higher concentration of GL or GB reveals the better anti-photoaging activity and GB has superior activity to GL.

**Fig. 6:**
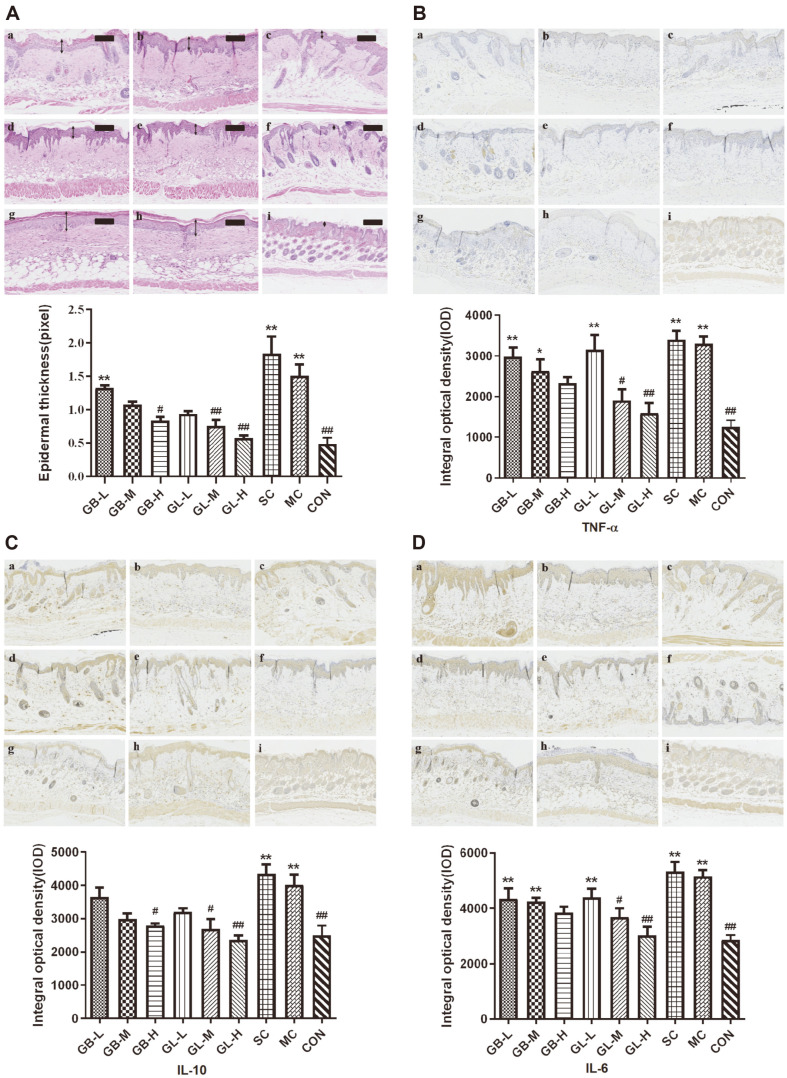
Influence of GB and GL on UV-B induced epidermal thickness. (**A**) Photographs of epidermal thickness observed by H&E staining (scale bars: 100 μm). The double-headed white arrows indicated the thickness of epidermis. The double-headed white arrows indicated the thickness of epidermis. Immunohistochemical staining of UV-B radiated mice skin for (**B**) TNF-α (**C**) IL-10 and (**D**) IL-6, (magnification × 200). (a) GB-L, (b) GB-M, (c) GB-H, (d) GL-L, (e) GL-M, (f) GL-H, (g) SC, (h) MC, and (i) CON. Histogram accompanied with error bar represents means ± SD (*n* = 3). Statistical significance indicated by *p < 0.05 significantly different from CON; #*p* < 0.05 significantly different from MC.

Epidermal hyperplasia is one of the characteristics of UV-B radiation induced skin lesions, and is often used as an indicator to evaluate the suppression of drugs on epidermal proliferation [32]. As the histological analysis of biopsied skin specimens is shown in Fig. 6, there is no significant difference in epidermal hyperplasia between MC and SC group, which are severer than the untreated control, furtherly confirmed that the solvent has no skin protective effect. However, the topical application of GL or GB can effectively suppress the epidermal proliferation. The higher concentration of GL or GB shows the better therapeutic effect. The skin thickness in Fig. 6 demonstrates that either GL or GB treatment reveals the significant reduction of skin thickness, comparing with MC and SC group. GL-H has the best skin protection from UVB radiation. Taken together, it also suggests that GL can inhibit epidermal proliferation induced by photoaging.

Because the production of tumor necrosis factor-α (TNF-α), interleukin (IL) and other inflammatory cytokines plays an important role in skin photoaging [33], thus immunohistochemical staining is used to analyze the influence of GB and GL on the formation of these key inflammatory cytokines. As shown in Fig. 6B and 6C, there is no significant difference in the formation of TNF-α, IL-6, and IL-10 between MC and SC group. More importantly, the production of TNF-α, IL-6, and IL-10 in mice treated by GL or GB solutions is significantly lower than that in MC and SC group. The GL-H treatment exhibits the most remark reduction in these inflammatory cytokines than other treatments.

## Conclusions

In this study, glabridin liposome (GL) is developed by film dispersion method to improve the inferior bioavailability of glabridin, and then its protection against UV-B radiation induced skin photoaging is also studied. The prepared GL has a spheroidal morphology with an average diameter of 200 nm. The GL has lower cytotoxicity than GB, but it exhibits a more effective role in the inhibition of melanin. Moreover, the application of GL can effectively remiss UVB radiation induced skin erythema and leathery skin, via inhibiting the production of inflammatory cytokines such as TNF-α, IL-6, and IL-10. Therefore, these results have demonstrated that GL has the potential as topical therapeutic materials against UVB radiation induced skin damage through inhibiting inflammation.
